# Design and Application of a Novel High-throughput Screening Technique for 1-Deoxynojirimycin

**DOI:** 10.1038/srep08563

**Published:** 2015-02-24

**Authors:** Peixia Jiang, Shanshan Mu, Heng Li, Youhai Li, Congmin Feng, Jian-Ming Jin, Shuang-Yan Tang

**Affiliations:** 1 CAS Key Laboratory of Microbial Physiological and Metabolic Engineering, Institute of Microbiology, Chinese Academy of Sciences, Beijing 100101, China; 2 Beijing Key Laboratory of Plant Resources Research and Development, Beijing Technology and Business University, Beijing 100048, China; 3 University of Chinese Academy of Sciences, Beijing 100049, China

## Abstract

High-throughput screening techniques for small molecules can find intensive applications in the studies of biosynthesis of these molecules. A sensitive, rapid and cost-effective technique that allows high-throughput screening of endogenous production of the natural iminosugar 1-deoxynojirimycin (1-DNJ), an α-glucosidase inhibitor relevant to the pharmaceutical industry, was developed in this study, based on the inhibitory effects of 1-DNJ on the activity of the β-glycosidase LacS from *Sulfolobus solfataricus*. This technique has been demonstrated effective in engineering both the key enzyme and the expression levels of enzymes in the 1-DNJ biosynthetic pathway from *Bacillus atrophaeus* cloned in *E. coli*. Higher biosynthetic efficiency was achieved using directed evolution strategies.

Directed evolution is a powerful tool frequently used in engineering the strains or biosynthetic pathways to improve the production of high-value small molecules^1^,^2^. The production of small molecules from the biosynthetic pathways can be impacted by the activities of key enzymes, the balance of the expression levels of pathway enzymes, and even the interactions of the host strain's native genetic network with the biosynthetic pathways^3^,^4^,^5^,^6^. Directed evolution of the biosynthetic pathways or the host genomes, is an efficient way to develop hyper-producing strains by optimization of the potential bottlenecks of the pathways to direct the carbon flux toward the desired products^7^,^8^. Elegant high-throughput screening techniques for small molecules can greatly aid the engineering processes^2^. The majority of small molecules are not ready to be easily detected with colorimetric or fluorescent screening techniques. Lack of proper screening techniques, especially *in vivo* screening techniques, has become the bottleneck in optimization of the biosynthetic pathways for higher yields of small-molecule products.

Iminosugars constitutes a group of sugar mimic alkaloids produced by plants or microorganisms as the secondary metabolites. As the eversible and competitive inhibitors of glycosidases, they are relevant to the pharmaceutical industry for the treatment of various diseases especially in the treatment of non-insulin-dependent (type II) diabetes^9^,^10^. 1-DNJ is one of the iminosugars. Known as α-glucosidase inhibitors, 1-DNJ and its derivatives have shown potential therapeutic effects on diabetes, HIV infection as well as Gaucher's disease^11^,^12^,^13^,^14^. 1-DNJ is mainly found in plants^15^, however, low amounts are found in some microorganisms^16^,^17^,^18^,^19^,^20^. The preparation of 1-DNJ include extraction from plants, microbial fermentation as well as chemical synthesis^21^. A combined biotechnological-chemical synthesis method is used for industrial production of 1-DNJ due to its short and economical process. The involved biotransformation step is the regioselective oxidation of 1-amino-1-deoxy-D-sorbitol to 6-amino-6-deoxy-L-sorbose, which is flanked by four chemical reactions^22^. Although the biosynthesis of 1-DNJ has attracted great interests, the studies are still preliminary. Genetic engineering and process optimization are still in great demand for improved 1-DNJ yield in microbial stains.

It has been demonstrated that the *TYB* gene cluster is responsible for catalysis of the first three steps of the 1-DNJ biosynthetic pathway in two *Bacillus* species. This cluster contains the *gabT1*, *yktc1* and *gutB1* genes, which encode a putative transaminase (GabT1), a phosphatase (Yktc1), and an oxidoreductase (GutB1), respectively^23^. The expression of the *TYB* gene cluster in *E. coli* led to 1-DNJ production. However, the genes encoding the epimerase and the reductase responsible for catalysis of the last two steps involving the conversion of mannojirimycin (MJ) to 1-DNJ remain unknown (Figure 1)^18^. Despite the report that MJ and 1-DNJ could be specifically assayed with α-mannosidase and trehalase inhibition reactions, respectively, these methods are laborious and time-consuming^16^. In order to engineer the biosynthesis of 1-DNJ for higher efficiency, a high-throughput screening technique for 1-DNJ is necessary. Here a solid phase-based sensitive high-throughput screening method for 1-DNJ was developed. The effectiveness of this method has been demonstrated through its applications in optimizing the *TYB* gene cluster for higher production of 1-DNJ.

## Results

### The development of 1-DNJ high-throughput screening technique

β-glycosidase from the archae *Sulfolobus solfataricus* (LacS) has broad substrate specificity and catalyzes the hydrolysis of aryl β-gluco, β-xylo and β-galactosides^24^. 1-DNJ was found able to inhibit LacS on its *o*-nitrophenyl-β-D-galactopyranoside (*o*NPG) hydrolysis activity (Figure 2a). The inhibitory effect of 1-DNJ on the LacS activity toward 5-bromo-4-chloro-3-indolyl β-D-galactopyranoside (X-GAL) was also demonstrated (Figure 2b). Five constructs expressing the *TYB* gene cluster (pDNJ1~5) were constructed. *E. coli* BWLacS was used as the expression host for all except pDNJ4 which was expressed in BL21(DE3) (Figure S1). Significant LacS inhibitory activity was detected in the cultures of BWLacS harboring pDNJ5, compared with the strain harboring a control plasmid without the gene cluster. This indicated that the inhibition was due to the products of the *TYB* gene cluster (Figure S2a).

The intermediate 2-amino-2-deoxy-D-mannitol (ADM) was found to not inhibit LacS (Figure S3). The effect of the unstable product of the *TYB* gene cluster, MJ (due to the unstable aminal functionality), on LacS activity was assayed with the reaction mixture of purified GutB1 with ADM and NAD^+^ as substrates^25^. MJ was also found to inhibit the activity of LacS (Figure S3). The production of MJ and 1-DNJ in strain BWLacS harboring pDNJ5 was also confirmed by the MJ-specific α-mannosidase assay and the 1-DNJ-specific trehalase assay (Figure S2b). The production of 1-DNJ was also confirmed by HPLC (Figure S4), NMR (Table S3) and ESI-MS.

**Figure 1:**
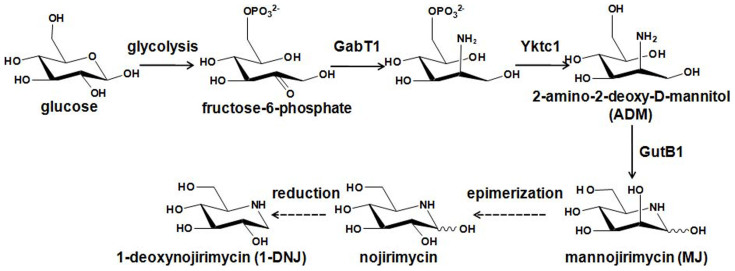
The proposed biosynthetic pathway of 1-DNJ, adapted from Horenstein^23^.

Based on the inhibitory effects of 1-DNJ, MJ and probably nojirimycin (Figure 1) on the activity of LacS, a solid-phase high-throughput screening method was developed. When cells coexpressing the *TYB* gene cluster and lacS were plated onto LB agar plates containing X-GAL, the cells producing higher titers of MJ and 1-DNJ were expected to show lighter blue (or whiter) color compared with lower producers. This high-throughput screening method was used to optimize the *TYB* gene cluster for higher production of 1-DNJ.

### Engneering of the key enzyme GutB1 in the *TYB* gene cluster

To explore the catalytic efficiency of the enzymes in the *TYB* gene cluster, random mutagenesis libraries of *gabT1*, *yktc1* and *gutB1* were constructed and transformed into strain BWLacS. The strains harboring the mutant libraries were grown for ~20 h on LB agar supplemented with 10 mM glucose as the precursor for 1-DNJ biosynthesis and X-GAL as substrate for LacS. Compared with BWLacS which harbored the wild-type *TYB* gene cluster and was grown under the same conditions, a higher ratio of light blue (or white) colonies was observed for GutB1 random mutagenesis library than for GabT1 or Yktc1 library, indicating that GutB1 plays a critical role in the biosynthetic efficiency of the pathway. Thus, we proceeded to use the GutB1 library for high-throughput screening. Eight whiter colonies of BWLacS harboring the GutB1 randomly mutated variants were selected (from a total of 10^4^ colonies screened), and the inhibitory effects of their LB liquid cultures on LacS activity were measured. Five mutant strains exhibited higher inhibitory effects on LacS activity toward *o*NPG, compared with the strain harboring the wild-type *TYB* gene cluster (Figure S5a). Plasmids from these clones were purified and retransformed into BWLacS, and the enhanced inhibitory activities on LacS were confirmed. Sequencing of the gutB1 genes in the five variants revealed three mutants (Gu2, Gu5 and Gu18) with one base-pair silent mutation (no change to amino acid sequence, the codon was changed to more frequently used codon in *E. coli*), mutant Gu1 and Gu30 with a D163G and an I236V substitutions, respectively (Table S4).

The results of the trehalase inhibition assay showed that, at 14 h, the 1-DNJ production of BWLacS harboring the I236V mutant was 54% higher than that of strain expressing wild-type GutB1 (Figure 3). A time course profile of MJ/1-DNJ production is presented in Figure S5b. It showed the inhibition rate of the culture on LacS activity upon *o*NPG and the cell growth (OD_600_) of BWLacS harboring either the I236V mutant or wild-type GutB1. The results showed that MJ/1-DNJ was produced in both the exponential and stationary phases. The supplemented glucose was almost exhausted in 3 h and led to a maximum inhibition rate of ~55% for the strain harboring wild-type GutB1 and 65% for strain harboring the I236V mutant.

### Characterization of the wild-type GutB1 and its I235V mutant

Wild-type GutB1^25^ and the I236V mutant were expressed in *E. coli* BL21(DE3) as N-terminal His-tagged fusion proteins and purified for an activity assay. D-sorbitol, D-mannitol and ADM were used as the substrates. Among the three substrates, wild-type GutB1 and the I236V mutant all showed the highest activities upon ADM, indicating that the amino group substitution at C2 of the hexitol chain of the substrate is preferred. The two enzymes also accepted D-sorbitol and D-mannitol as substrates albeit at reduced rates. For all three substrates, the I236V mutant showed ~2-fold higher specific activity compared with wild-type GutB1 (Figure 4). The results of kinetic studies revealed that the I236V mutant exhibited higher affinity for the substrate NAD^+^ than the wild-type enzyme. For both substrates, *k_cat_* of the I236V mutant was higher than that of the wild-type enzyme. Therefore, the catalytic efficiency of I236V mutant was higher than that of the wild-type enzyme for both substrates (Table 1).

### Engineering of the expression levels of the enzymes in the *TYB* gene cluster

It has been reported that fine-tuning the expression levels of individual genes in the biosynthetic pathway to optimize the metabolic flux is critical for the production of the final metabolites of the pathway^26^. To explore the effects of the expression levels of individual genes in the *TYB* gene cluster on the production of MJ/1-DNJ, the nucleotides in the RBS regions of *yktc1* and *gutB1* were randomized simultaneously (Figure S6). This RBS library was subjected to high-throughput screening. Twelve whiter clones were selected (from a total of 8 × 10^5^ mutants screened) and rescreened in liquid LB. Nine of them exhibited higher inhibitory activities on LacS toward *o*NPG compared with the strain carrying the wild-type *TYB* gene cluster (Figure S7a). Plasmids from these clones were purified and retransformed into strain BWLacS and the improved inhibitory activities on LacS were confirmed. Sequencing the mutated *TYB* gene clusters in clone M12 and M13 revealed the mutated RBS regions (Table S5). At 14 h, the production of 1-DNJ from strain BWLacS carrying pM13 was 68% higher than that of the strain carrying pDNJ6, as determined using the trehalase inhibition assay (Figure 3).

The time courses of MJ/1-DNJ production are presented in Figure S7b. They showed the inhibition rate of the culture on LacS activity toward *o*NPG, as well as the cell growth of the BWLacS carrying pM13 or pDNJ6. The maximum inhibition rate reached ~73% for pM13 and ~62% for pDNJ6.

### Characterization of the wild-type and RBS-mutated *TYB* gene clusters

To measure the expression levels of Yktc1 and GutB1 from BWLacS harboring pDNJ6 or pM13, sfGFP was fused to the C-terminal of GutB1 or Yktc1 (Figure S8). The sfGFP fluorescence, representing the expression levels of Yktc1 or GutB1 in both plasmids, was measured (Figure 5). The expression level of GutB1 in pM13 was slightly higher than that in pDNJ6, while the expression level of Yktc1 in pM13 was ~1/3 of that in pDNJ6. This suggests that a lower ratio of Yktc1 to GutB1 expression led to a higher production of MJ/1-DNJ. These results demonstrate that an appropriate ratio of pathway enzyme expression levels, rather than the high expression of all of them, leads to greater biosynthetic efficiency.

## Discussion

We have demonstrated the design and application of a novel high-throughput screening technique for endogenous 1-DNJ production in this study. 1-DNJ was mainly found as an inhibitor of α-glucosidases, rarely as a β-glucosidase or galactosidase inhibitor^27^. For example, it does not have inhibitory effect on the β-galactosidase from *E. coli* (LacZ). In this study, 1-DNJ was found able to inhibit LacS, a β-glycosidase with broad substrate specificity. Therefore, a blue-white screening technique developed based on the inhibition of 1-DNJ on the hydrolysis activity of LacS on X-GAL was designed and successfully applied for the high-throughput screening of endogeneous 1-DNJ production on agar plates.

It has been demonstrated that the production of 1-DNJ could be improved by either elevating the activity of the rate-limiting enzyme GutB1 or balancing the relative expression levels of the pathway enzymes. The results from these two studies all revealed that higher activity of GutB1 relative to Yktc1 was helpful for 1-DNJ production improvement, indicating again that GutB1 catalyzes the rate-limiting step in the wild-type biosynthetic pathway. After the optimization of the biosynthetic pathway, the rate-limiting step may change and further additional improvements of 1-DNJ is foreseeable by identification and engineering of the rate-limiting enzymes, optimization of the genetic networks of the host strain for better supply of the biosynthetic precursors and cofactors, as well as optimization of process fermentation.

**Figure 2:**
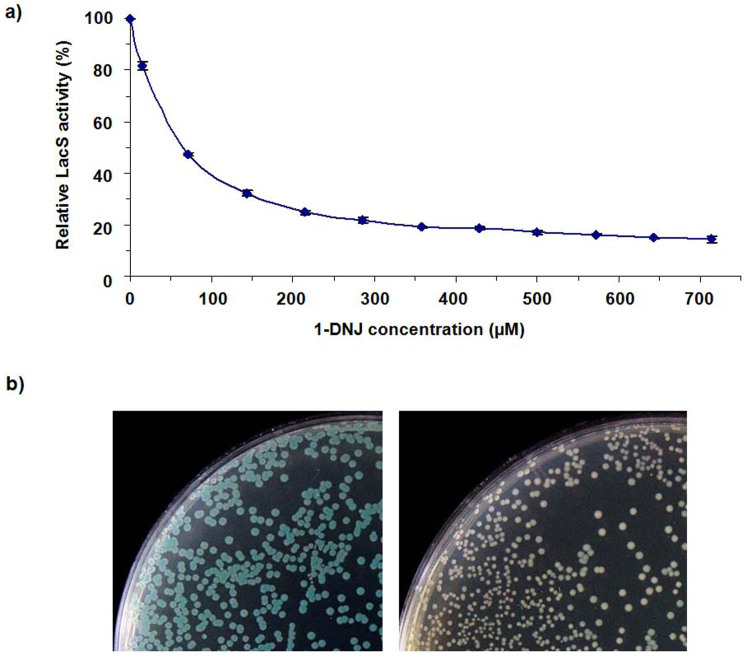
Inhibition of LacS activity by 1-DNJ. (a) *In vitro* inhibition of *o*NPG hydrolysis. (b) *In vivo* inhibition of X-GAL hydrolysis. Strain BWLacS expressing LacS was grown on LB agar supplemented with 1 mM L-arabinose, 40 μg·mL^−1^ X-GAL and 0 (left) or 0.5 mM (right) 1-DNJ.

**Figure 3:**
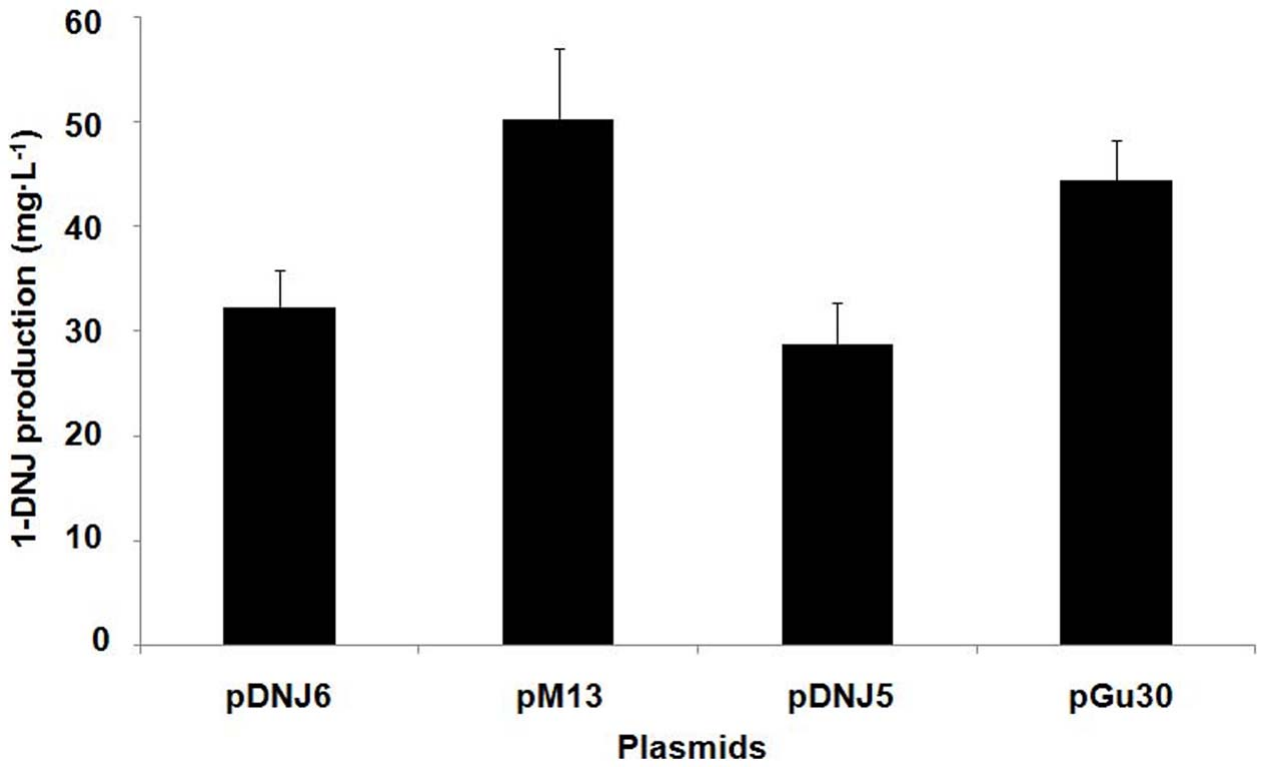
The 1-DNJ production of strain BWLacS harboring various plasmids cultured for 14 h.

From Figure 2, the inhibitory effect of 1-DNJ on LacS showed high sensitivity at low concentrations of 1-DNJ. Therefore, the current screening system may not be suitable for screening when the productivity of 1-DNJ is relatively high because the colonies may all appear white on the agar plates. Then a LacS mutant which is less sensitive to the 1-DNJ inhibition needs to be developed. The problem may also be partly solved by engineering the rate-limiting enzyme in a lower-producing strain and then put the evolved mutant enzyme back into the higher-producing strain.

Due to the broad substrate specificity of LacS, it was also found to be inhibited by MJ, and probably by other iminosugars of similar structures as 1-DNJ, which will further broaden the application of this high-throughput screening technique. This technique also has potential applications in the screening of inhibitors of glycosidases.

## Methods

### General

Restriction enzymes were purchased from New England Biolabs (Beijing, China) and DNA polymerases were purchased from Takara Bio Inc. (Dalian, China). T4 DNA ligase was purchased from Life Technologies (Shanghai, China). Oligonucleotides and the promoter P_cp6_^28^ were synthesized by Life Technologies (Shanghai, China). 1-Deoxynojirimycin (1-DNJ) was purchased from Carbosynth Limited (Berkshire, UK). D-mannosamine hydrochloride, α-mannosidase (from *Canavalia ensiformis*), 4-nitrophenyl-α-D-mannoside, trehalose and trehalase (from porcine kidney) were purchased from Sigma-Aldrich (St. Louis, USA). The strain *Bacillus atrophaeus* (Agricultural Culture Collection of China, ACCC 02297) was purchased as a 1-DNJ-producing strain.

All bacteria were routinely grown in Luria-Bertani (LB) medium. The antibiotics ampicillin (100 μg·mL^−1^), kanamycin (50 μg·mL^−1^) and apramycin (50 μg·mL^−1^) were used when necessary.

### Strain Construction

The β-glycosidase gene *lacS* (Genbank accession No. AF133096.1) was amplified with the genomic DNA of *Sulfolobus solfataricus* (kindly provided by Prof. Li Huang from Institute of Microbiology, Chinese Academy of Sciences) as template using primers LacS-for-NdeI and LacS-rev-XhoI, and the PCR product was ligated into vector pBAD18 after digestion with NdeI and XhoI, resulting in plasmid pBADLacS. The DNA fragment containing *araC* and *lacS* under control of promoter P_BAD_ was PCR-amplified with plasmid pBADLacS as template using primers AraC-LacS-XbaI-for and LacS-rev-XhoI. This fragment was then ligated into the PCR product amplified using primers pAH-for-XbaI and pAH-rev-XhoI with the CRIM plamid pAH156^29^ as template after digestion with XbaI and XhoI, resulting in a construct in which *lacS* is under control of P_BAD_ promoter, adjacent to the gentamycin resistance gene and flanked by regions homologous to the *E. coli* HK022 integration site. The construct was integrated into the *E. coli* BW25113 chromosome using helper plasmid pAH69 as described^29^, resulting in strain BWLacS. The integration was verified by PCR.

### Library Construction

MEGAWHOP-PCR^30^ was performed for library construction as follows:

1. Error-Prone PCR Library. The random mutagenesis libraries of GabT1, Yktc1 and GutB1 were constructed through error-prone PCR. Primer pairs gabT1-for/gabT1-rev, yktc1-for/yktc1-rev, gutB1-for/gutB1-rev were used to amplify the *gabT1*, *yktc1* and *gutB1*, respectively, using pDNJ5 as template. The PCR reaction mixture consisted of 5 mM MgCl_2_, 0.2 mM each of dATP and dGTP, 1 mM each of dCTP and dTTP, 0.1 mM MnCl_2_ and rTaq DNA polymerase. Then the PCR products obtained, containing randomly mutated *gabT1*, *yktc1* and *gutB1* genes, were used as the megaprimers to perform the MEGAWHOP PCR using pDNJ5 as template, respectively. Following the MEGAWHOP PCR, DpnI digestion (20 U) of the template was performed at 37°C for 2 h, then DpnI was inactivated at 80°C for 20 min. The PCR products were transformed into *E. coli* MC1061 and around 5 × 10^4^ transformants were recovered for each library (random mutagenesis libraries of GabT1, Yktc1 or GutB1). Ten randomly picked clones from each library were sequenced and contained an average of 3.6, 2.5 and 2.8 nucleotide mutations per clone for GabT1, Yktc1 and GutB1 libraries, respectively. All colonies from the agar plates for each library were used for plasmid isolation to prepare the plasmid library.
2. RBS Library. Primers Lib-for-RBS1 and Lib-rev-RBS2 were designed with degenerate sequences at the RBS regions of *yktc1* and *gutB1* to produce the diverse changes tailored for exploring a vast sequence space of RBS strengths^31^ (Figure S6). The DNA fragment containing the two RBS regions was generated by PCR using the primers Lib-for-RBS1 and Lib-rev-RBS2 with pDNJ6 as template using Pyrobest DNA polymerase. Then the PCR product was used as megaprimer to perform MEGAWHOP PCR using pDNJ6 as template. After DpnI digestion, the MEGAWHOP PCR product was transformed into *E. coli* MC1061 and 1 × 10^6^ transformants were recovered. Ten randomly picked clones were sequenced, and these sequences revealed the expected random mutations at the targeted nucleotide positions, with no additional point mutations. All colonies from the agar plates were used for plasmid isolation to prepare the plasmid library.

### High-throughput screening of the random mutagenesis library and RBS library

The plasmid libraries of random mutagenesis and RBS libraries were transformed into strain *E. coli* BWlacS, respectively. Cells were plated onto LB agar plates containing 1 mM L-arabinose, 0.1 mM IPTG, 10 mM glucose (as the substrate for 1-DNJ production), 40 μg·mL^−1^ X-Gal and 100 μg·mL^−1^ ampicillin. The plates were incubated at 37°C for 14 h, and the colonies which were obviously whiter (by the eye) than those carrying wild-type plasmid pDNJ5 (for random mutagenesis library screening) or pDNJ6 (for RBS library screening) were picked for quantifying DNJ production in liquid culture.

### Preparation of samples for 1-DNJ/MJ inhibition assays

A single colony of strain BWLacS harboring plasmid carrying wild-type or mutant *TYB* gene cluster was grown in 3 mL of LB containing ampicillin for 12 h at 37°C. The cultures were then diluted to OD_600_ = 0.1 in 20 mL of LB and 0.5 mM IPTG was added to the culture when OD_600_ reached 0.8 at 37°C to induce the expression of the *TYB* gene cluster. The culture was grown at 30°C for 2 h after induction and then 10 mM glucose was added to the culture as the substrate for 1-DNJ synthesis. Samples were withdrawn at 0–26 h (the time point of IPTG addition was regarded as 0 h) during the fermentation. The samples were centrifuged and the supernatant was taken for the downstream inhibition assays.

### β-Glycosidase (LacS) inhibition assay

The reaction mixture for the LacS inhibition assay was prepared by the addition of 5 μL of 1-DNJ standard solution, enzyme reaction mixture or the culture supernatant, 645 μL buffer Z and 40 μL of 12 mM *o*NPG. Finally, 2.5 μg of purified LacS was added into the mixture. After incubation at 37°C for 10 min, the reaction was terminated by the addition of 185 μL of 1 M sodium carbonate and the absorbance at 420 nm was measured with a SynergyMx Multi-Mode Microplate Reader (BioTek, Vermont, USA). 5 μL Buffer Z instead of the sample was used in the control experiment. One unit of LacS enzyme activity was defined as the amount of enzyme required to liberate the equivalent of 1 μmole of *o*-nitrophenol per min under the assay conditions. The LacS inhibition rate was calculated as follows: 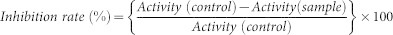


All reported data in Figures 2a, S2a, S3, S5 and S7 represent the mean of three independent data points. The error bars represent standard deviations.

### Enzymatic assays for GutB1

The enzymatic activity of GutB1 and its I236V mutant was monitored by following NAD^+^ reduction at 340 nm. The reaction mixture (200 μL) contained 100 mM Tris-HCl buffer (pH 8.5), 25 mM NaCl, 0.25 mM ZnCl_2_, 2 mM NAD^+^, certain amount of purified GutB1 or its I236V mutant enzyme (1.3 μg for ADM reaction and 5.1 μg for sorbitol and mannitol reactions), as well as 5 mM ADM or 50 mM D-sorbitol or D-mannitol as the substrate. The reactions were initiated by the addition of NAD^+^ and incubated at 30°C for 10 min. The increase of absorbance at 340 nm was measured with a SynergyMx Multi-Mode Microplate Reader (BioTek, Vermont, USA). Background NAD^+^ reduction in the absence of enzyme was subtracted.

**Figure 4:**
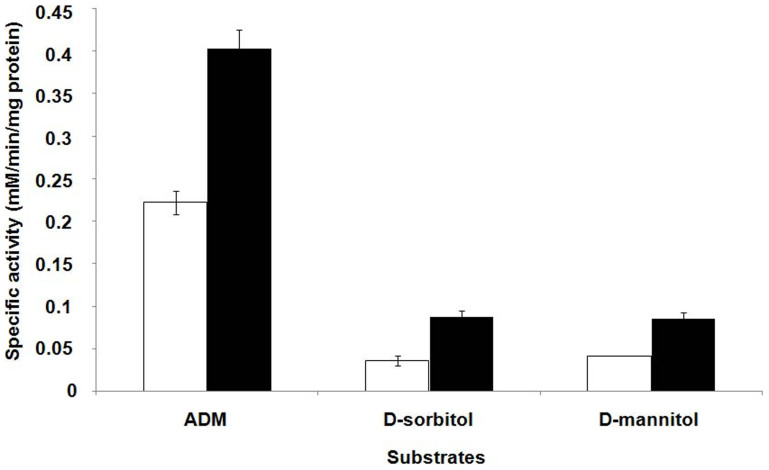
The specific activity of wild type (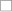) and mutant (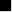) GutB1 on various substrates.

All reported data in Figure 4 represent the mean of three independent data points. The error bars represent standard deviations.

### Kinetic analysis of wild-type and mutant GutB1

0.02 ~ 5 mM of ADM (0.5 mM NAD^+^) or 0.05 ~ 1 mM of NAD^+^ (0.5 mM ADM) were used in determining the kinetic parameters of GutB1 wild-type and I236V mutant enzymes with the method described above. All assays were performed in three replicates and the kinetic parameters in Table 1 were obtained using Lineweaver-Burk plots. All reported data in Table 1 represent the mean of three independent data points.

### Fluorescence assay

A colony of BWLacS cells harboring plasmid pDNJ6-yktc-sfgfp, pM13-yktc-sfgfp, pDNJ6-gutB-sfgfp or pM13-gutB-sfgfp was grown overnight at 37°C in LB medium containing ampicillin, then diluted to OD_600_ = 0.8 in the same medium containing 0.5 mM IPTG, and allowed to grow at 30°C for 12 h. A total of 200 μL of culture was centrifuged, and the cells were washed with 10 mM potassium phosphate buffer (pH 7.4) and resuspended in 200 μL of the same buffer. The cell suspension optical density (OD_600_) and fluorescence emission were measured with a SynergyMx Multi-Mode Microplate Reader (BioTek, Vermont, USA) (485 nm excitation filter, 528/20 nm emission filter). The background fluorescence due to buffer served as the blank in all measurements.

**Figure 5:**
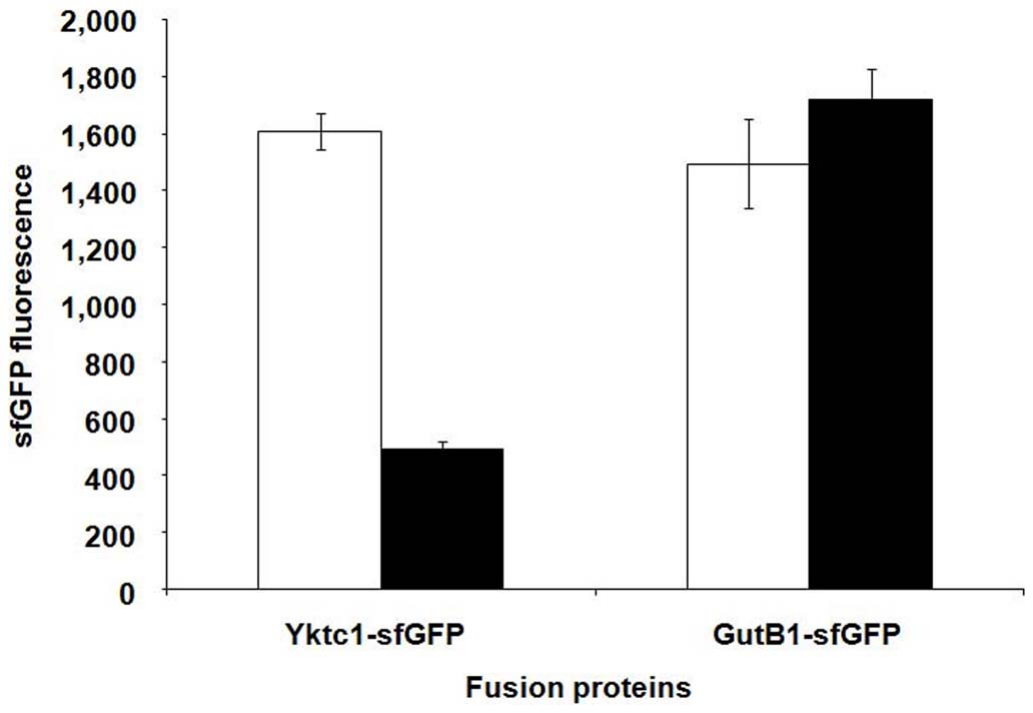
The sfGFP fluorescence measured from strain BWLacS expressing sfGFP-fused Yktc1 or GutB1 in both wild-type (pDNJ6, 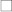) and mutant (pM13, 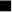) *TYB* gene clusters.

All reported data in Figures 5 represent the mean of three independent data points. The error bars represent standard deviations.

## Author Contributions

P.J., S.M., H.L., Y.L. and C.F., performed the experiments and analyzed the data. S.-Y.T. and J.-M.J., planned the experiments and wrote the manuscript. All authors reviewed the manuscript.

## Supplementary Material

Supplementary Information supplementary information

## Figures and Tables

**Table 1:** Kinetic parameters of the wild-type GutB1 and its I236V mutant for substrates ADM and NAD^+^

Enzymes_(substrate)_	*K_m_* (mM)	*k_cat_* (min^−1^)	*k_cat_/K_m_* (mM^−1^ min^−1^)
Wild-type_(ADM)_	0.063 ± 0.005	0.25 ± 0.005	4.0
I236V_(ADM)_	0.064 ± 0.007	0.48 ± 0.02	7.5
Wild-type_(NAD_^+^_)_	0.10 ± 0.008	0.41 ± 0.02	4.1
I236V_(NAD_^+^_)_	0.054 ± 0.002	0.50 ± 0.01	9.3
