# Supplementary material for: Design and Application of a Novel High-throughput Screening Technique for 1-Deoxynojirimycin
Source: Sci Rep. 2015 Feb 24;5:8563. doi: 10.1038/srep08563 (PMC4338435; doi:10.1038/srep08563)
Supplement: Supplementary Information [file srep08563-s1.pdf]

Supplementary Information for:

Design and Application of a Novel High-throughput Screening Technique for 1-Deoxynojirimycin

Peixia Jiang[1]*, Shanshan Mu[1,3]*, Heng Li[1,3], Youhai Li[1], Congmin Feng[1], Jian-Ming Jin[2], Shuang-Yan Tang[1]

[1]CAS Key Laboratory of Microbial Physiological and Metabolic Engineering, Institute of Microbiology, Chinese Academy of Sciences, Beijing 100101, China, [2]Beijing Key Laboratory of Plant Resources Research and Development, Beijing Technology and Business University, Beijing 100048, China, [3]University of Chinese Academy of Sciences, Beijing 100049, China.

Methods

Plasmid Construction. All strains and plasmids used in this study are shown in Table S1. Sequences for all primers are listed in Table S2. Promoter P_{cp6}[1] was amplified with primers Cp6-PstI-for and Cp6-XbaI-rev and ligated into plasmid pDHK29[2] after digestion of PstI and XbaI, resulting in plasmid pDHK29-Pcp6. The *bla* gene (ampicillin resistance marker) in vector pFLAG-CTC (Sigma-Aldrich, St. Louis, USA) was replaced with the *aac* gene expressing apramycin resistance, resulting in plasmid pLAC5. The *TYB* gene cluster was amplified using *B. atrophaeus* genomic DNA as the template with primers gabT1-for- NdeI and gutB1-SacI-rev designed based on the published nucleotide sequence of strain *B. atrophaeus* (GenBank accession No. CP002207.1).[3] The 3258-bp PCR product was ligated into vectors pBAD18, pDHK29-Pcp6, pLAC5, pET28a and pMALp2X after digestion of NdeI and SacI, resulting in plasmids pDNJ1, pDNJ2, pDNJ3, pDNJ4 and pDNJ5, respectively (Figure S1).

In the native *TYB* gene cluster, the 3' regions of *gabT1* and *yktc1* were overlapped with the 5' regions of *yktc1* and *gutB1*, respectively (Figure S6). To study the expression of each gene, two RBSs were designed and placed upstream of *yktc1*and *gutB1*, respectively. pDNJ6 was constructed based on plasmid pDNJ5. The procedure of the construction was as follows: the *gabT1*, *yktc1* and *gutB1* gene fragments were amplified using pDNJ5 as template with the primer pairs gabT1-NdeI-for/PRBS1-rev, PRBS1-for/PRBS2-rev and PRBS2-for/gutB1-SacI-rev, respectively. Equimolar aliquots of these three PCR products (0.2 pmole for each) obtained above were PCR-assembled without primers. Finally outer primers gabT1-NdeI-for and gutB1-SacI-rev were added to the assembly reaction and the entire gene cluster was PCR-amplified.[4] The PCR product was ligated into pMALp2X after digestion with NdeI and SacI, resulting in plasmid pDNJ6, in which two RBSs were placed upstream of *yktc1* and *gutB1*, respectively (Figure S1, Figure S6).

To measure the expression levels of *yktc1* and *gutB1* genes in plasmid pDNJ6 and pM13, SuperFolder green fluorescent protein (sfGFP), a robustly folded version of

GFP,[5] was fused to the C-terminal of Yktc1 and GutB1, respectively, in both pDNJ6 and pM13 (Figure S8). The *sfgfp* gene was kindly provided by Prof. Chunbo Lou from Institute of Microbiology, Chinese Academy of Sciences. Plasmids pDNJ6-yktc-sfgfp and pM13-yktc-sfgfp were constructed by fusing sfGFP to the C-terminal of Yktc1 in plasmids pDNJ6 and pM13, respectively. The procedure of plasmid constructions was as follows: The DNA fragments F1 and M13-F1 containing *gabT1* and *yktc1* were amplified using the primer pair gabT1-for-AseI/Yktcgfp-rev with pDNJ6 and pM13 as template, respectively. The DNA fragments F2 and M13-F2 containing *sfgfp* were amplified using primer pairs sfgfp-for/sfgfp-rev and sfgfp-for/sfgfp-M13-rev, respectively. The DNA fragments F3 and M13-F3 containing *gutB1* were amplified with pDNJ6 and pM13 as templates using primer pairs Yktcgfp-for-WT/gutB1-SacI-rev and Yktcgfp-for-M13/gutB1-SacI-rev, respectively. Equimolar (0.1 pmole) aliquots of the three DNA fragments in each group (F1/F2/F3 and M13-F1/M13-F2/M13-F3) were PCR-assembled without primers. Then the outer primers gabT1-for-AseI and gutB1-SacI-rev were added to the assembly reaction and the gene cluster with *sfgfp*-fused *yktc1* was PCR-amplified. The overlapped PCR products from the two groups of DNA fragments were ligated into pMALp2X after digestion of AseI and SacI, resulting in plasmids pDNJ6-yktc-sfgfp and pM13-yktc-sfgfp, respectively (Figure S8).

pDNJ6-gutB-sfgfp and pM13-gutB-sfgfp were constructed by fusing sfGFP to the C-terminal of GutB1 in plasmid pDNJ6 and pM13, respectively. The procedure of plasmid construction was as follows: The DNA fragments F_{DNJ} and M13-F_{DNJ} containing the *TYB* gene cluster were amplified using the primer pair gabT1-for-AseI/gutbgfp-rev with pDNJ6 and pM13 as templates, respectively. The DNA fragment sfgfp was amplified using the primers sfgfp-for and sfgfp-rev-SacI. Equimolar aliquots (0.1 pmole) of the two purified DNA fragments in each group (F_{DNJ}/sfgfp and M13-F_{DNJ}/sfgfp) were PCR-assembled and then amplified with primers gabT1-for-AseI and sfgfp-rev-SacI. The overlapped PCR products from the two groups of DNA fragments were ligated into pMALp2X after digestion with AseI and SacI, resulting in plasmids pDNJ6-gutB-sfgfp and pM13-gutB-sfgfp, respectively

(Figure S8).

The PCR product of *lacS* obtained in the "strain construction " section was ligated into vector pET28a after digestion with NdeI and XhoI, resulting in plasmid pET28a-lacS.

Purification of 6-His tagged LacS. *E. coli* BL21 (DE3) were used as the host strain for the expression and purification of LacS protein. A single colony of strain *E. coli* BL21 (DE3) harboring pET28-lacS was grown in 3 ml of LB containing kanamycin at 37 °C for 12 h. The culture was then diluted to OD_{600}=0.1 and grown at 37 °C and 0.5 mM IPTG was added when OD_{600} reached 0.8, then the culture was continuously grown at 30 °C for 10 h. Cells were harvested by centrifugation at 4 °C at 3000 ×g for 20 min. The cells were then resuspended in 10 ml of buffer Z (40 mM NaH_2PO_4, 60 mM Na_2HPO_4, 10 mM KCl, 1 mM $MgSO_4$, 50 mM β-mercaptoethanol, pH 8.0) and lysed by sonication. The lysate was heated at 75 °C for 10 min and centrifuged at 15000 ×g for 30 min at 4 °C. The supernatant was applied to Ni-NTA resin. The column was washed with 20 mM imidazole in buffer Z and the bound protein was eluted with 200mM imidazole in buffer Z. The imidazole was removed by dialysis at 4 °C against buffer Z. The purity of proteins were assessed with SDS-PAGE and the protein concentration was assayed with Bradford method.[6]

Trehalase inhibition assay for 1-DNJ. 1-DNJ production was monitored by inhibition of trehalase activity as described previously[7] with modifications. 80 μL 1-DNJ standard solution or the boiled culture supernatant was added to 120 μL of 100 mM maleate buffer (pH 6.0) containing 3.85 mU trehalase, and the mixture was incubated at 37 °C for 15 min. Then 10 μL of 56 mM trehalose in 100 mM maleate buffer (pH 6.0) was added and the reaction mixture was incubated at 37 °C for a further 60 min. The production of glucose was quantified with the 3,5-dinitrosalicylic acid (DNS) method.[8] One unit of trehalase enzyme activity was defined as the amount of enzyme required to convert 1.0 μmole of trehalose to 2.0 μmoles of glucose per

min under the assay condition. The trehalase inhibition rate was calculated as described in the "β-glycosidase inhibition assay" section. The concentration of 1-DNJ in the supernatant was calculated with a calibration curve prepared with 1-DNJ standard solutions (0.02-0.2 mM).

All reported data in Figures 3 and S2b represent the mean of three independent data points. The error bars represent standard deviations.

α-Mannosidase inhibition assay. MJ production was monitored by inhibition of mannosidase activity according to the method by Hardick.[7] 10 μL of The GutB1 reaction mixture or 1-DNJ standard solution was incubated with 3 μg α-mannosidase in 250 μL of 100 mM sodium acetate buffer (pH 5.5) at 25 ^{o}C for 15 min. 50 μL of 15 mM 4-Nitrophenyl-α-D-mannoside was added and the reaction mixture was further incubated at 25 ^{o}C for 15 min. The reaction was stopped by addition of 200 μL of 0.1 M glycine-NaOH buffer (pH 10.7) and the absorbance at 400 nm was determined. 10 μL of 100 mM Tris-HCl buffer (pH 8.5) instead of the sample was used in the control experiment. One unit of α-mannosidase enzyme activity was defined as the amount of enzyme required to hydrolyze 1 μmole of 4-nitrophenyl-α-D-mannoside in 1 min under the assay condition. The α-mannosidase inhibition rate was calculated as described in the "β-glycosidase inhibition assay" section.

All reported data in Figures S2b represent the mean of three independent data points. The error bars represent standard deviations.

Purification of 6-His tagged GutB1 and its I236V mutant. The *gutB1* and its I236V mutant genes were amplified using the primer pair gutB1-for-NdeI/gutB1-rev-XhoI with pDNJ5 and pI236V as templates, respectively. The PCR products were ligated into pET28a after digestion with NdeI and XhoI, resulting in plasmids pET28a-gutB1 and pET28a-I236V, respectively. A single colony of strain *E. coli* BL21 (DE3) harboring pET28a-gutB1 or pET28a-I236V was grown in 3 mL of LB containing kanamycin at 37 ^{o}C for 12 h. The culture was then diluted to OD_{600}=0.1 and grown at 37 ^{o}C and 0.5 mM IPTG was added when OD_{600} reached 0.8, then the culture was

continuously grown at 30 °C for 10 h. Cells were harvested by centrifugation at 4 °C at 3000 ×g for 20 min. The cells were then resuspended in 10 mL of 20 mM Tris-HCl buffer (pH 8.0, 250 mM NaCl, 5 mM imidazole) and lysed by sonication with a JY92-IIN Ultra Sonic Cell Crusher (Ningbo, China). The lysate was centrifuged at 15000 ×g for 30 min at 4 °C and the supernatant was applied to Ni-NTA resin (Sigma-Aldrich, St. Louis, USA). The column was washed with 20 mM Tris-HCl buffer (pH 8.0, 250 mM NaCl, 50 mM imidazole) and the bound protein was eluted with 20 mM Tris-HCl buffer (pH 8.0, 250 mM NaCl, 250 mM imidazole). The imidazole was removed by dialysis at 4 °C against 20 mM Tris-HCl buffer (pH 8.0, 25 mM NaCl). The purity of proteins were assessed by sodium dodecyl sulfate polyacrylamide gel electrophoresis (SDS-PAGE) and the protein concentration was assayed with Bradford method.[6]

Glucose quantification. The residual glucose in the culture was quantified with DNS method.[8]

Preparation of 2-amino-2-deoxy-D-mannitol (ADM). ADM was prepared by $NaBH_4$ reduction of D-mannosamine hydrochloride as described previously[9-10] with some modifications. 2 mL D-Mannosamine hydrochloride (172 mg) was incubated with 4.5 mL of 3.5 % $NaBH_4$ (in NH_4OH) for 1 h at room temperature. Then acetic acid was carefully added to remove the excess $NaBH_4$. Methanol was added to the quenched reaction mixture and then the reaction mixture was concentrated under reduced pressure. This process was repeated five times. 2 mL H_2O was added to the concentrated reaction mixture and the pH was adjusted to ~5.0. The mixture was deionized by an Amberlite IR120 resin (H^+ form) column and then applied to a silica gel column (0.5 g). The column was washed with ethyl acetate-methanol-water-1 % HCl (16:4:1:1). Then ADM was eluted with ethyl acetate-methanol-water-1 % HCl (14:6:1:1). 128 mg of ADM (as the HCl salt) was obtained.

Purification and identification of 1-DNJ. 3 L of the culture of strain BWLacS

harboring pDNJ5 prepared as described in "preparation of samples for 1-DNJ/MJ inhibition assays" section and grown for 14 h after glucose addition was centrifuged and the supernatant was loaded on Amberlite IR120 resin (H^+ form) column. The column was washed with 2.5 bed volumes of deionized water and then eluted with 0.5 M NH_4OH. The fractions containing 1-DNJ (detected by LacS inhibitory assay) were collected and loaded on Dowex 1×2 resin (OH^- form). The column was washed with the deionized water. Again, the active fractions were collected and dried under reduced pressure. The active sample (4.0 g) was repeatedly chromatographed on silica gel column with the gradient ethyl acetate-methanol- ammonia water (from 8:2:1 to 5:5:1) to give 22 mg of 1-DNJ. NMR spectra were recorded on a Bruker Avance III 400 MHz instrument (400 MHz for ^1H NMR, 100 MHz for ^{13}C NMR at 25 °C) (Fallanden, Switzerland) (Table S3)[11]. MS was recorded on an Agilent 6520 LC/MS instrument (Agilent Technologies, Santa Clara, USA). ESI-MS spectrum of the purified 1-DNJ showed a molecular ion peak $[M+H]^+$ at *m/z* 164 (base peak).

HPLC determination of 1-DNJ. The supernatant obtained above was loaded on Amberlite IR120 resin (H^+ form) (Sigma-Aldrich, St. Louis, USA). The column was washed with 2.5 bed volumes of deionized water. Then the column was eluted with 2.5 bed volumes of 0.5 M NH_4OH and the fractions containing 1-DNJ (verified by LacS inhibitory assay) were combined and loaded on Dowex 1×2 resin (OH^- form) (Sigma-Aldrich, St. Louis, USA). The column was eluted with 2.5 bed volumes of deionized water. Again, the active fractions were collected and concentrated to 1 mL. 1-DNJ in the samples were labeled using 9-fluorenylmethyl chloroformate (FMOC-Cl) and then analyzed using HPLC.[12-13] 20 μL of 10 mM FMOC-Cl in CH_3CN was added to the mixture of 10 μL 1-DNJ sample with 10 μL 0.4 M potassium borate buffer (pH 8.5) by immediate mixing and allowed to react at 20 °C for 20 min. Then 10 μL of 0.1 M glycine was added to terminate the reaction by quenching the remaining FMOC-Cl. To stabilize the 1-DNJ-FMOC, 950 μL of 0.1 % acetic acid was added into the reactant. HPLC was performed using Shimadzu LC-20A system (Shimadzu Corp., Kyoto, Japan) equipped with a SPD-M20A photodiode array (PDA) detector

operating at 254 nm. Separation was achieved using a Waters Symmetry C18 column (250×4.6 mm, 5 μm) (Waters, USA) working at 30 ^{o}C with the mobile phase of acetonitrile: 0.1 % acetic acid (1:1, v/v) at a flow rate of 0.6 mL·min^{-1} (Figure S4).

References:

1 Jensen, P. R. & Hammer, K. The sequence of spacers between the consensus sequences modulates the strength of prokaryotic promoters. *Appl. Environ. Microbiol.* **64**, 82-87 (1998).

2 Phillips, G. J., Park, S. K. & Huber, D. High copy number plasmids compatible with commonly used cloning vectors. *Biotechniques* **28**, 400-402, 404, 406 passim (2000).

3 Hardicka, D. J. & Hutctdnsunb, D. W. The Biosynthesis of 1-deoxynojirimycin in *Bacillus subtilis* var niger. *Tetrahedron* **49**, 6707-6716 (1993).

4 An, Y. *et al.* A rapid and efficient method for multiple-site mutagenesis with a modified overlap extension PCR. *Appl. Microbiol. Biotechnol.* **68**, 774-778 (2005).

5 Pedelacq, J. D., Cabantous, S., Tran, T., Terwilliger, T. C. & Waldo, G. S. Engineering and characterization of a superfolder green fluorescent protein. *Nat. Biotechnol.* **24**, 79-88 (2006).

6 Bradford, M. M. A rapid and sensitive method for the quantitation of microgram quantities of protein utilizing the principle of protein-dye binding. *Anal. Biochem.* **72**, 248-254 (1976).

7 Hardick, D. J., Hutchinson, D. W., J.TreWa, S. & Wellingtot, E. M. H. Glucose is a precursor of 1-deoxynojirimycin and 1-deoxymannonojirimycin in *Streptumyces subrutilus*. *Tetrahedron* **48**, 6285-6296 (1992).

8 Miller, G. L. Use of dinitrosalicylic acid reagent for determination of reducing sugar. *Anal. Chem.* **31**, 426-428 (1959).

9 Liu, T. Y., Gotschlich, E. C., Dunne, F. T. & Jonssen, E. K. Studies on the meningococcal polysaccharides. II. Composition and chemical properties of the group B and group C polysaccharide. *J. Biol. Chem.* **246**, 4703-4712 (1971).

10 Wu, Y., Arciola, J. & Horenstein, N. Medium-chain dehydrogenases with new specificity: amino mannitol dehydrogenases on the azasugar biosynthetic pathway. *Protein Pept. Lett.* **21**, 10-14 (2014).

11 Zhu, Y. P. *et al.* Purification and identification of 1-deoxynojirimycin (DNJ) in okara fermented by *Bacillus subtilis* B2 from Chinese traditional food (Meitaoza). *J. Agr. Food Chem.* **58**, 4097-4103 (2010).

12 Kim, J. W. *et al.* Determination of 1-deoxynojirimycin in *Morus alba* L. leaves by derivatization with 9-fluorenylmethyl chloroformate followed by reversed-phase high-performance liquid chromatography. *J. Chromatogr. A* **1002**, 93-99 (2003).

13 Kang, K. D. *et al.* Identification of the genes involved in 1-deoxynojirimycin synthesis in *Bacillus subtilis* MORI 3K-85. *J. Microbiol.* **49**, 431-440 (2011).

14 Datsenko, K. A. & Wanner, B. L. One-step inactivation of chromosomal genes in *Escherichia coli* K-12 using PCR products. *Proc. Natl. Acad. Sci. USA* **97**, 6640-6645 (2000).

Figures and Tables

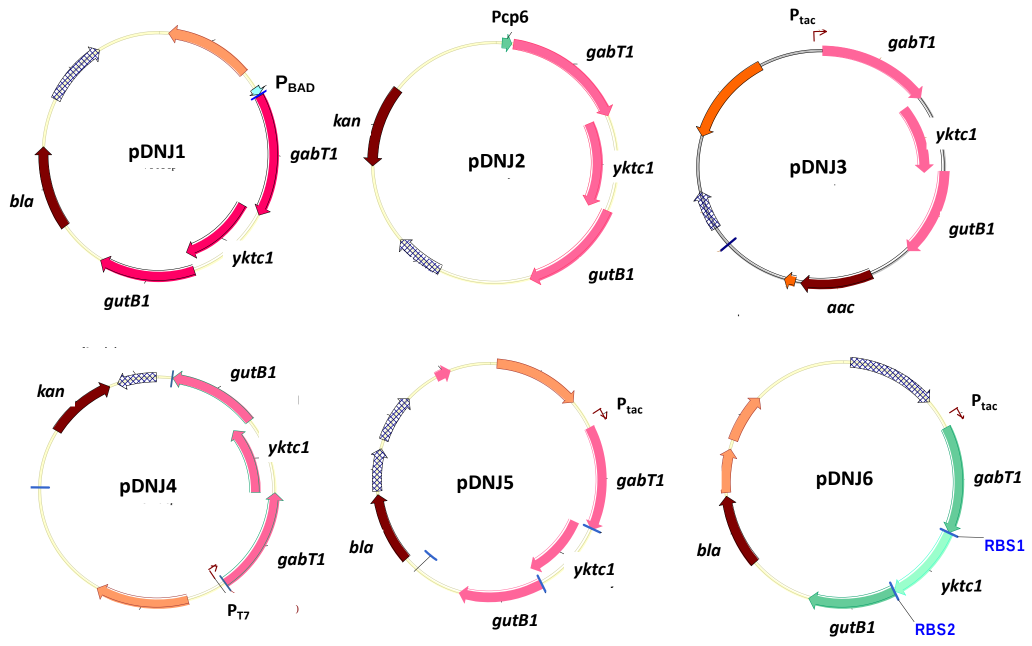

Figure S1 | Plasmid maps of pDNJ1~6

a)

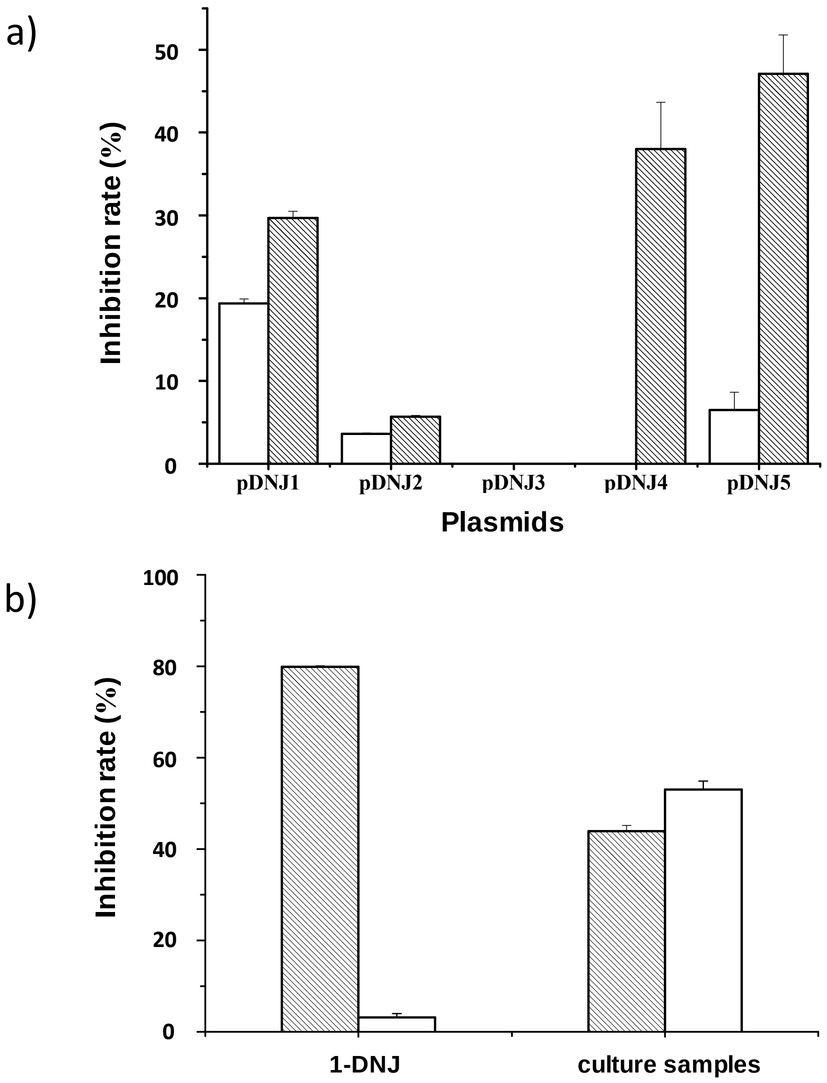

Figure S2 | The inhibitory effects of the cultures of strains expressing the *TYB* gene cluster on LacS, trehalase and α-mannosidase. (a) The inhibition rates of LacS activity on *o*NPG hydrolysis by the LB culture of stains harboring plasmid pDNJ1~5 (slash) compared with those of strains harboring the corresponding control plasmids not containing the *TYB* gene cluster (open). *E. coli* BWLacS was used as the expression host for all the plasmids except pDNJ4 which was expressed in BL21(DE3). (b) The inhibition rates of trehalase activity (slash) and α-mannosidase activity (open) by 1-DNJ standard solution (0.38 mM) and the LB culture of stain BWLacS harboring plasmid pDNJ5 cultured for 14 h.

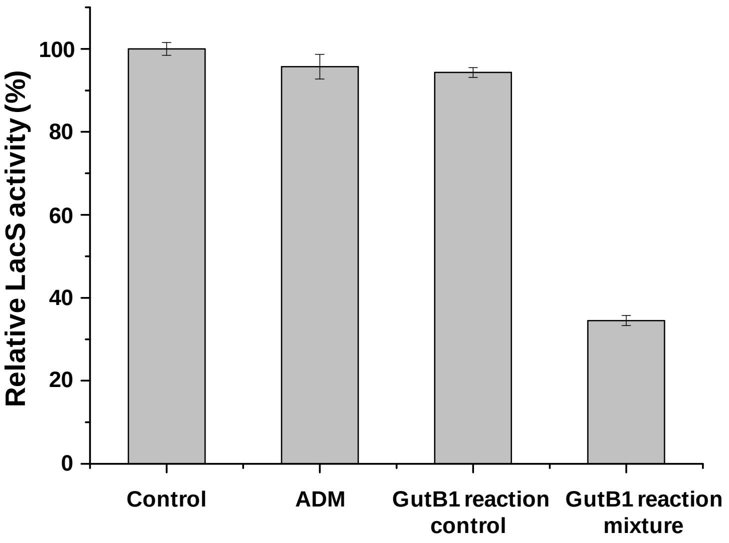

Figure S3 | The inhibitory effects of ADM (25 mM) and MJ on LacS activity toward *o*NPG, using buffer Z as the control. Due to the instability of MJ, its inhibitory assay was done with 10 μL of the following reaction mixture: 400 μL of 100 mM Tris-HCl buffer (pH 8.5) containing 25 mM NaCl, 0.25 mM $ZnCl_2$, 9 mM NAD^+, 25 mM ADM and 320 μg of purified GutB1 incubated at 37 ^oC for 4 h. The reaction mixture in the absence of GutB1 was used as a control.

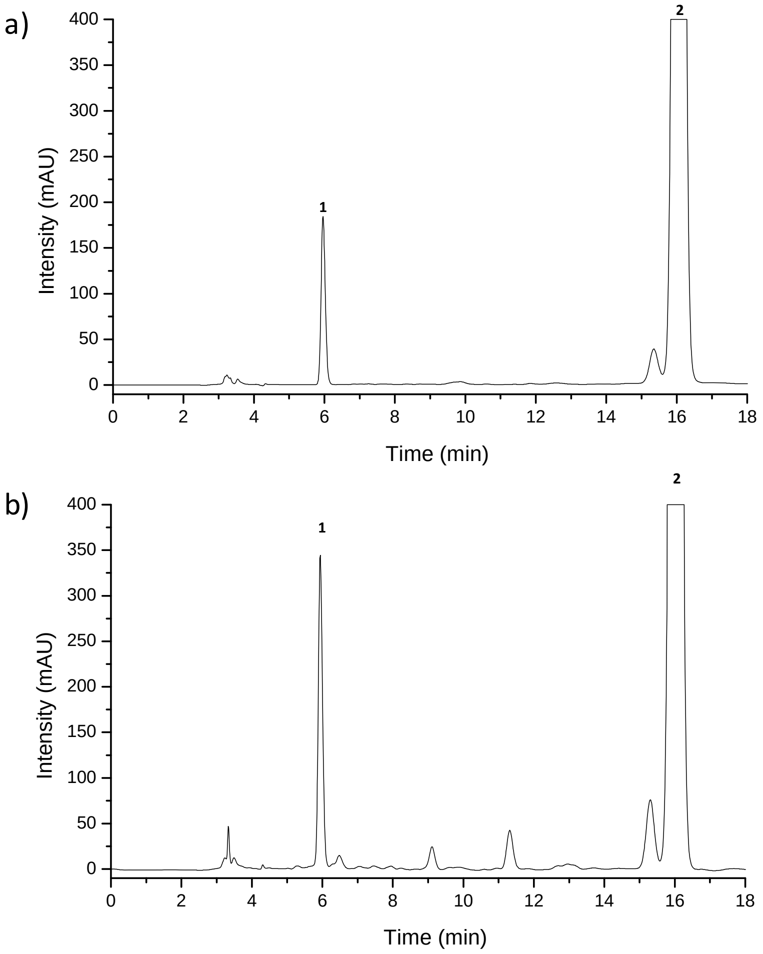

Figure S4 | HPLC chromatograph of 1 mg·mL^{-1} 1-DNJ standard solution (a) and 1-DNJ produced in the culture of strain BWLacS harboring pDNJ5 (b). Peak 1. 1-DNJ- FMOC-Cl; peak 2. glycine- FMOC-Cl.

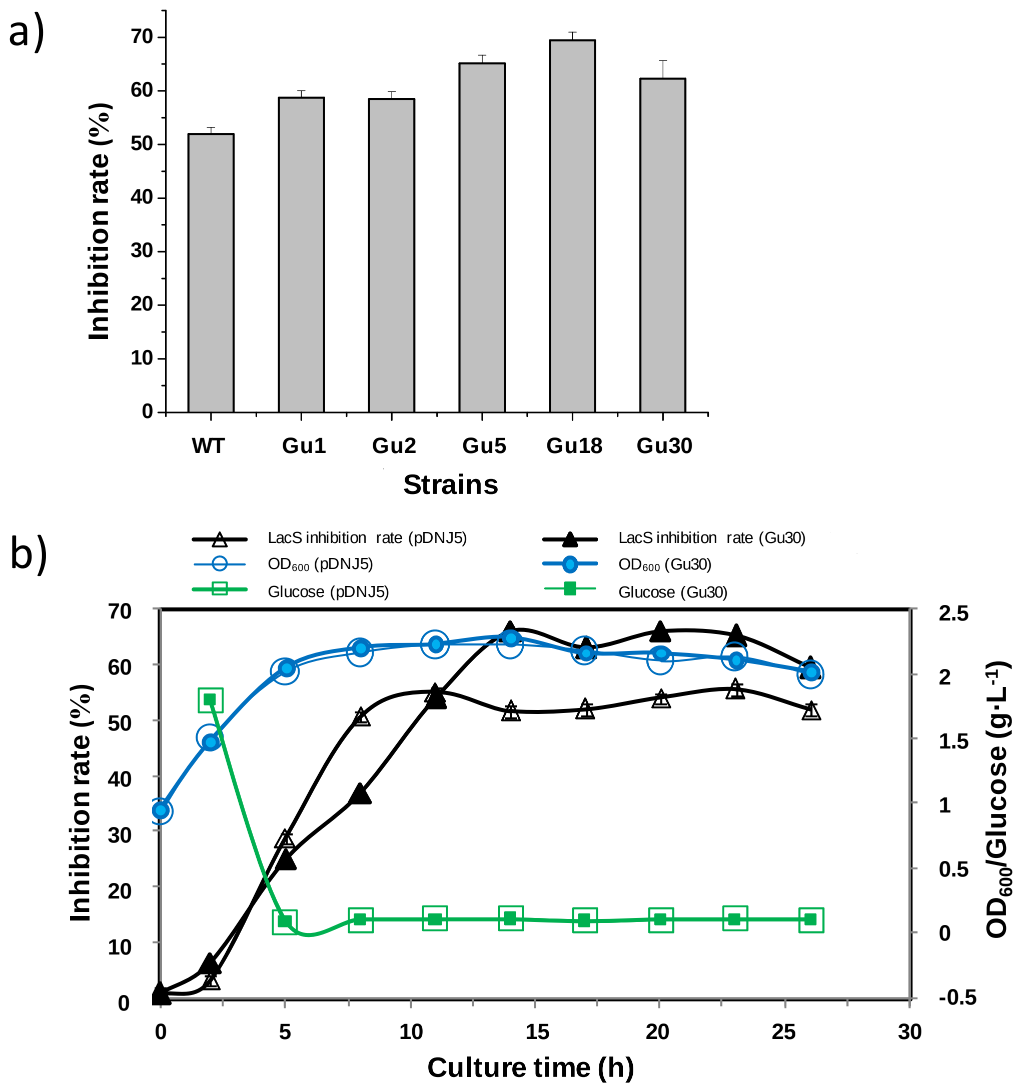

Figure S5 | Screening and characterization of GutB1 mutants. (a) Mutant strains exhibiting higher inhibition rates on LacS activity toward *o*NPG screened from the random mutagenesis library of GutB1. (b) Time course profiles of the inhibition rate on LacS activity toward *o*NPG, cell growth (OD_{600}) and amount of residual glucose measured from the culture of wild-type and Gu30 mutant strains.

gabT1*-RBS1-*yktC1

pDNJ5 TATAAAGTGAGAGACTATATTA

⇩ native gene cluster

pDNJ6 TATAAAG**TAA**TAAT**aggagg**TGACTG**ATG**AGAGACTATATTA

⇩ gene cluster with designed RBS

RBS library TATAAAG**TAA**TAA**DRRRRRDD**GACTG**ATG**AGAGACTATATTA

gene cluster with degenerate RBS (D=A,G,T; R=A,G)

yktC1*-RBS2-*gutB1

pDNJ5

AGAAAGGAAGGGAGCT**ATG**AAGGCGTTGGTCTGGACTCCTAA

⇩ native gene cluster

pDNJ6

AGAAAGGAAGGGAGCTACGAAGGCGTTGGTCTGGACTCCTAATAAT**aggagg**TTGAT
T**ATG**AAGGCGTTGGTCTGGACTCCTAA

⇩ gene cluster with designed RBS

RBS library

AGAAAGGAAGGGAGCTACGAAGGCGTTGGTCTGGACTCCTAATAA**DRRRRRDD**TG
ATT**ATG**AAGGCGTTGGTCTGGACTCCTAA

gene cluster with degenerate RBS (D=A,G,T; R=A,G)

Figure S6 | RBS optimization strategy. In the native *TYB* gene cluster (pDNJ5), the 3' regions of *gabT1* and *yktc1*were overlapped with the 5' regions of *yktc1* and *gutB1*, respectively. RBSs were designed and placed upstream of *yktc1* and *gutB1*, resulting in the modified *TYB* gene cluster in pDNJ6. The two RBS regions were degenerated simultaneously to construct the RBS library.

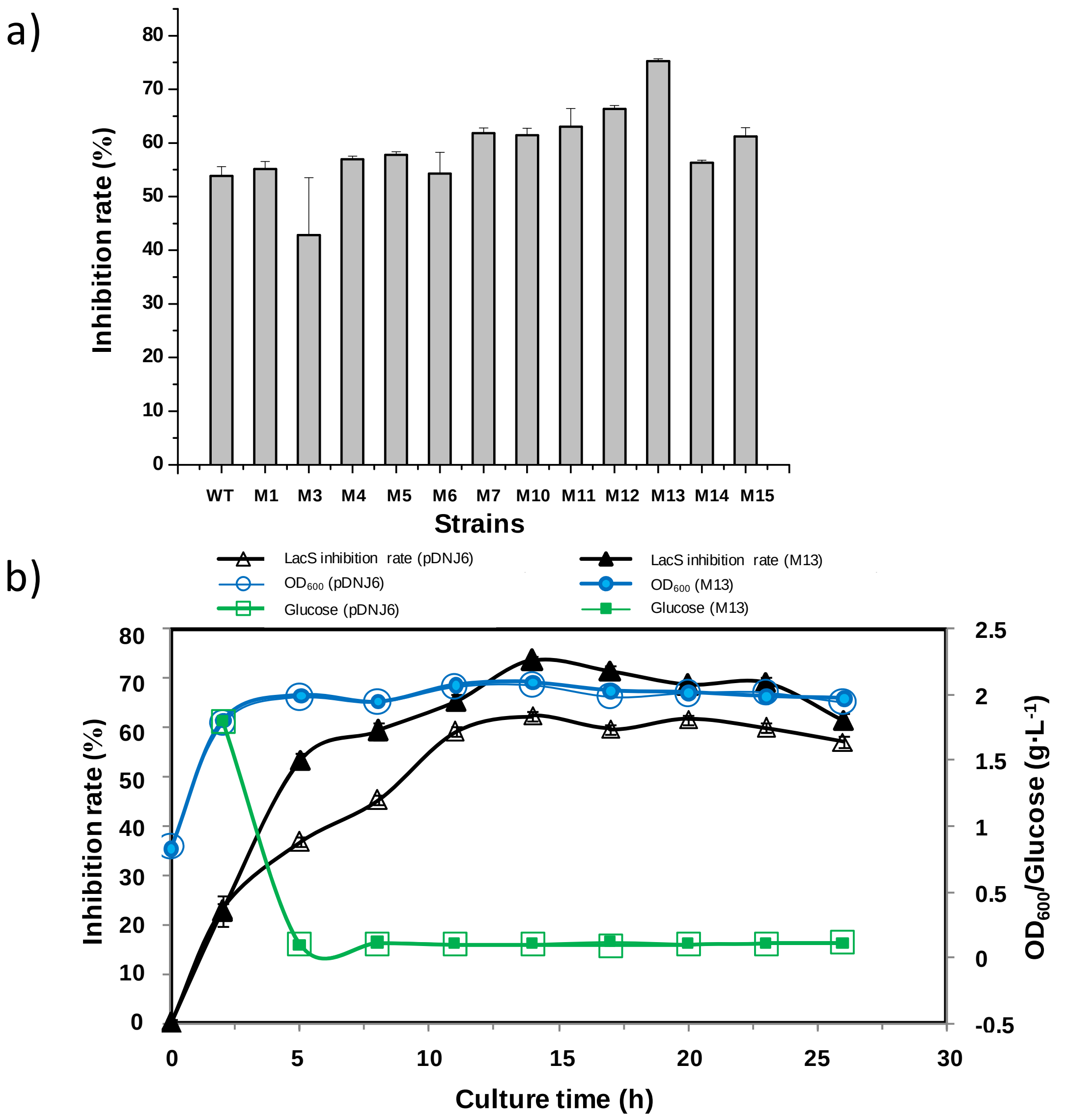

Figure S7 | Screening and characterization of mutants from RBS library. (a) Mutant strains exhibiting higher inhibition rates on LacS activity toward *o*NPG screened from the RBS library. (b) Time course profiles of the inhibition rate on LacS activity toward *o*NPG, cell growth (OD_{600}) and amount of residual glucose measured from the culture of wild-type and M13 mutant strains.

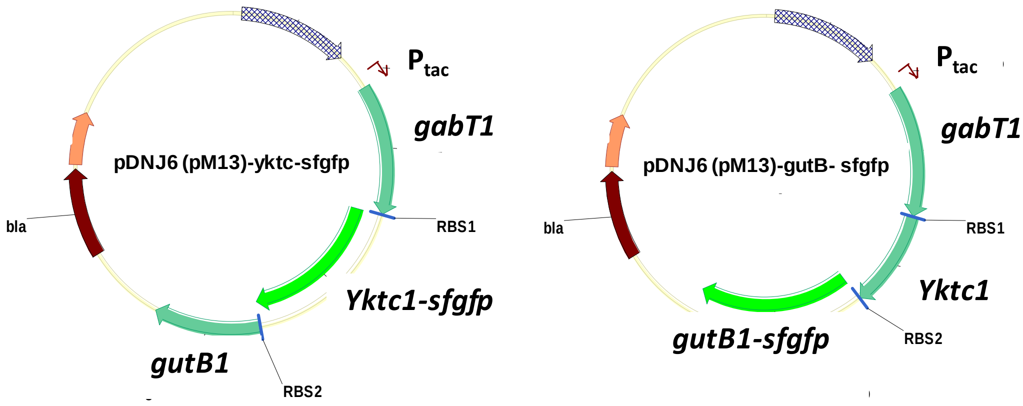

Figure S8 | Plasmid maps of pDNJ6 (pM13)-yktc-sfgfp and pDNJ6 (pM13)-gutB-sfgfp. sfGFP was fused to the C-terminus of Yktc1 or GutB1 in both pDNJ6 and pM13.

Table S1 | Strains and plasmids used in this study

Resource	Relevant characteristics	Source
**Strains**		
B. atrophaeus		ACCC
BW25113		[14]
BWLacS	BW25113 with the integration of P_{BAD}-*lacS*	This work
**Plasmids**		
pBADLacS	*lacS* under the control of promoter P_{BAD}	This work
pDNJ1	*TYB* gene cluster under the control of promoter P_{BAD}	This work
pDHK29-Pcp6	Promoter Pcp6 cloned into pDHK29	This work
pDNJ2	*TYB* gene cluster under the control of promoter Pcp6	This work
pLAC5	*bla* gene in pFLAG-CTC was replaced with the *aac* gene	This work
pDNJ3	*TYB* gene cluster under the control of promoter P_{tac}	This work
pDNJ4	*TYB* gene cluster under the control of T7 promoter	This work
pDNJ5	*TYB* gene cluster under the control of promoter P_{lac}	This work
pGu30	mutated *TYB* gene cluster in pDNJ5 carrying the I236V mutant of *gutB1*	This work
pDNJ6	*TYB* gene cluster under the control of promoter P_{lac}, an RBS placed upstream of each of the three genes.	This work
pM13	mutated *TYB* gene cluster in pDNJ6 with the RBS regions mutated	This work
pDNJ6-yktc-sfgfp	*yktc1-sfgfp* fusion gene in the *TYB* gene cluster	This work
pM13-yktc-sfgfp	*yktc1-sfgfp* fusion gene in the RBS-mutated *TYB* gene cluster	This work
pDNJ6-gutB-sfgfp	*gutB1-sfgfp* fusion gene in the *TYB* gene cluster	This work
pM13-gutB-sfgfp	*gutB1-sfgfp* fusion gene in the RBS-mutated *TYB* gene cluster	This work
pET28a-gutB1	*gutB1* under the control of T7 promoter	This work
pET28a-I236V	the I236V mutant of *gutB1* under the control of T7 promoter	This work
pET28a-lacS	*lacS* under the control of T7 promoter	This work

Table S2 | Primers used in this study

Primers	Sequences [a]
gabT1-NdeI-for	5'-TA***CATATG***GGGACGAAGGAAATTACAAA -3'
gutB1-SacI-rev	5'-TAT***GAGCTC***TTACACCAGCTTCGGATCAGATAC-3'
Cp6-PstI-for	5'-ATT***CTGCAG***GATCCCATTATGCATGTGGGA-3'
Cp6-XbaI-rev	5'-CCT***TCTAGA***CCCATTCTTAAGGATCCC-3'
PRBS1-for	5'-TAATAATAGGAGGTGACTGATGAGAGACTATATTATTG-3'
PRBS1-rev	5'-TCATCAGTCACCTCCTATTATTACTTTATATCCTCCAATAACTT-3'
PRBS2-for	5'-TAATAGGAGGTTGATTATGAAGGCGTTGGTCTGGA -3'
PRBS2-rev	5'-CTTCATAATCAACCTCCTATTATTAGGAGTCCAGACCAACGCCTTCGTAGCTCCCT TCCTTTCTGT-3'
Lib-for-RBS1	5'-GGATATAAAGTAATAADRRRRRDDGACTGATGAGAGACTATATTA-3' D=A,G,T; R=A,G
Lib-rev-RBS2	5'-ACCAACGCCTTCATAATCAHHYYYYYHTTATTAGGAGTCCA-3' H=A,C,T; Y=C,T
LacS-for-NdeI	5'- AGA***CATATG***TACTCAT TTCCAAATAG CTT -3'
LacS-rev-XhoI	5'-GGT***CTCGAG***TTAGTGCCTTAATGGCT-3'
AraC-LacS-XbaI-for	5'-TCCG***TCTAGA***CGTCAATTGTCTGATTCGTTACCA-3'
pAH-for-XbaI	5'-AAAG***TCTAGA*** ATTCTTGAAG ACACTAGAAT TGTGA-3'
pAH-rev-XhoI	5'-TACCGAG***CTCGAG***TTCTCATGTTTGAC-3'
gabT1-for	5'-ATGGGGACGAAGGAAATTACAAA-3'
gabT1-rev	5'- TCCCCATTCCAATAATATAGTCTCTCA-3'
yktc1-for	5'- CTGGGACAGAAAGGAAGGGAGCTATG-3'
yktc1-rev	5'- TGTTATTGTTGTTGTTGTTCGAGCTCTTA-3'
gutB1-for	5'-ATGAAGGCGTTGGTCTGGACT-3'
gutB1-rev	5'-TTACACCAGCTTCGGATCAGA-3'
gutB1-for-NdeI	5'- AT***CATATG***AAGGCGTTGGTCTGGACTCCTAA
gutB1-rev-XhoI	5'-TAT***CTCGAG***TTACACCAGCTTCGGATCAGATAC-3'
sfgfp-for	5'-GGCGGCGGTGGCGGTCGTAAAGGCGAA-3'

sfgfp-rev	5'-TCATAATCAACCTCCTATTATTATTTGTACAGTTCATCCA TACCATGCGTGAT-3'
sfgfp-M13-rev	5'-TCATAATCATCTTCCCTTTATTATTTGTACAGTTCATCCA TACCATGCGTGAT-3'
gabT1-for-AseI	5'-ATAG***ATTAAT***GGGGACGAAGGAAATTACAAA-3'
Yktcgfp-rev	5'-TTCGCCTTTACGACCGCCACCGCCGCCGGAGTCCAGACCAACGCCTTCGTA-3'
Yktcgfp-for-WT	5'-TAATAATAGGAGGTTGATTATGAA-3'
Yktcgfp-for-M13	5'-TAATAAAGGGAAGATGATTATGAA-3'
gutBgfp-rev	5'-TTCGCCTTTACGACCGCCACCGCCGCCCACCAGCTTCGGATCAGATACAA GGA-3'
sfgfp-rev-SacI	5'-ATTC***GAGCTC***TCATTTGTACAGTTCATCCATACCAT-3'

[a] Restriction sites were indicated in italics and underlined.

Table S3 | ^{13}C and ^{1}H NMR data of the isolated 1-DNJ

Position	δ_C	δ_H
1	48.7 (t)	3.59 (t, *J*= 12.4, 5.2 Hz), 3.06 (t, *J*= 12.4, 11.6 Hz)
2	69.7 (d)	3.88 (m)
3	78.9 (d)	3.60 (t, *J*= 9.2, 9.2 Hz)
4	70.6 (d)	3.68 (t, *J*= 9.6, 10.4 Hz)
5	62.8 (d)	3.18 (m, H-5)
6	60.5 (t)	4.02 (dd, *J*=12.8, 3.2 Hz), 3.95 (dd, *J*=12.8, 5.2 Hz)

*Spectra were measured at 100 MHz for ^{13}C and 400 MHz for ^{1}H in D_2O.

Table S4 | Amino acid substitutions in GutB1 mutants

Variants	Amino acid substitutions
Gu1	D163G
Gu2	T339T (silent mutation)
Gu5	D27D (silent mutation)
Gu18	D27D (silent mutation)
Gu30	I236V

Table S5 | Ribosomal binding site (RBS) sequences upstream of *yktc1* (RBS-1) and *gutB1* (RBS-2) in *TYB* gene cluster

Variants	RBS-1 (TAADRRRRRDDG)	RBS-2(TAADRRRRRDDT)
Wild-type	TAATAGGAGGTG	TAATAGGAGGTT
M12	TAATAAGAGGAG	TAATAGGAGGTT
M13	TAATGAAGAGGG	TAAAGGGAAGAT
